# Thyroid Hormone Signaling and Function: News from Classical and Emerging Models

**DOI:** 10.3390/cells11030453

**Published:** 2022-01-28

**Authors:** Maria Sirakov, Michelina Plateroti

**Affiliations:** 1 Department of Biology and Evolution of Marine Organisms, Stazione Zoologica Anton Dohrn, Villa Comunale, 80121 Naples, Italy; maria.sirakov@szn.it; 2 Team: Development, Cancer and Stem Cells, Université de Strasbourg, Inserm, Interface de Recherche Fondamentale et Appliquée en Cancérologie (IRFAC)/Unité Mixte de Recherche (UMR)-S1113, Fédération de Médecine Translationnelle de Strasbourg (FMTS), 67200 Strasbourg, France

According to Brown and Cai, Thyroid hormones (THs) have been considered “the first developmental morphogen ever discovered” [1]. This assumption arises from the very first observation of their capability to trigger amphibian metamorphosis at the beginning of the 20th Century [2]. From that point, a long journey started and investigations pointed out the pleiotropic role played by THs in regulating developmental as well as homeostatic processes in several vertebrates’ organs. The classical model of amphibian metamorphosis remains a fundamental example of THs multivarious role as morphogen, since the increase in circulating THs can induce at the same time in different organs apoptosis together with growth and neo-morphogenesis [3]. The mechanisms by which THs can control these very diverse developmental programs depending on the cell type and tissue is still puzzling and still actively investigated. Indeed, to better understand this diversity we need to take into account different elements involved in this signaling pathway, some of them conserved also in invertebrate taxa. There are three key components that have also been identified in a few non vertebrate taxa. In vertebrates the thyroid peroxidase (TPO) synthesizes the pro-hormone L-thyroxine using iodine and tyrosine as precursors. Thyroid gland releases THs, namely L-thyroxine (T4) and triiodothyronine (T3), which act at a cellular level. The selenoprotein iodothyronine deiodinases (DIO) are also important actors of intracellular activity for THs, since they locally convert T4 into T3 or rT3 (reverse triiodothyronine) or T3 in T2, thus regulating T3 availability [4]. Finally, genomic and non-genomic TH actions depend upon thyroid hormone nuclear receptors (TRs), which are T3-modulated transcription factors, able to bind DNA regions named Thyroid hormone Responsive Elements (TREs) in order to regulate the transcription of target genes [5].

This article collection highlights the role of THs in different tissues, taking into account data from classical and novel models. The work of Esposito et al., gave an insight on the evolution of THs signalling comparing the homologies of the available sequences of TPO, DIOs, and THRs. Thus, their study supports the hypothesis of the evolutionary adaptation of a functional thyroid hormone signaling in non-vertebrate chordates [6]. Nittoli et al. evaluate the TH disrupting activity of pesticides, in the context of testicular toxicity comparing mouse and zebrafish. In particular, the authors proposed the possibility to use zebrafish as a suitable model to go in depth and dissect such responses [7]. The review from Zekri et al., provides an interesting summary of THs role in controlling adaptive thermogenesis (i.e., high energy expenditure and low body mass index) pointing out the debate of local versus central control of THs activity [8]. Finally, the classical model of amphibian metamorphosis is still shown to be a fundamental model to apply to new techniques, as well as new biological questions. Indeed, looking at *Xenopus tropicalis* metamorphosis, both the cross-talk of THs and glucocorticoids and the intestinal morphogenesis are explored using state-of-the-art functional genomic and developmental biology approaches by Buisine [9] and Shibata [10], respectively.

Throughout these articles the roles and mechanisms of action of THs are covered in different fields of interest including non-mammalian models. Taken together, these contributions should be of wide interest for both scientists of fundamental fields and physicians for translational approaches.
